# Bioimpedance Analysis as Early Predictor for Clot Formation Inside a Blood-Perfused Test Chamber: Proof of Concept Using an In Vitro Test-Circuit

**DOI:** 10.3390/bios13030394

**Published:** 2023-03-17

**Authors:** Muhammet Türkmen, Tobias Lauwigi, Tamara Fechter, Fabienne Gries, Anna Fischbach, Thomas Gries, Rolf Rossaint, Christian Bleilevens, Patrick Winnersbach

**Affiliations:** 1 Department of Anesthesiology, University Hospital RWTH Aachen, Pauwelsstraße 30, 52074 Aachen, Germany; 2 Institut für Textiltechnik (ITA), RWTH Aachen University, 52074 Aachen, Germany

**Keywords:** bioimpedance analysis, clot detection, sensor fibers, membrane oxygenator, in vitro test circuit, coagulation

## Abstract

Clot formation inside a membrane oxygenator (MO) due to blood-to-foreign surface interaction represents a frequent complication during extracorporeal membrane oxygenation. Since current standard monitoring methods of coagulation status inside the MO fail to detect clot formation at an early stage, reliable sensors for early clot detection are in demand to reduce associated complications and adverse events. Bioimpedance analysis offers a monitoring concept by integrating sensor fibers into the MO. Herein, the feasibility of clot detection via bioimpedance analysis is evaluated. A custom-made test chamber with integrated titanium fibers acting as sensors was perfused with heparinized human whole blood in an in vitro test circuit until clot formation occurred. The clot detection capability of bioimpedance analysis was directly compared to the pressure difference across the test chamber (ΔP-TC), analogous to the measurement across MOs (ΔP-MO), the clinical gold standard for clot detection. We found that bioimpedance measurement increased significantly 8 min prior to a significant increase in ΔP-TC, indicating fulminant clot formation. Experiments without clot formation resulted in a lack of increase in bioimpedance or ΔP-TC. This study shows that clot detection via bioimpedance analysis under flow conditions in a blood-perfused test chamber is generally feasible, thus paving the way for further investigation.

## 1. Introduction

In the past two decades, the use of extracorporeal membrane oxygenation (ECMO) for acute respiratory distress syndrome (ARDS) increased substantially, especially during pandemics of viral respiratory infections such as the 2009 influenza H1N1 and the ongoing SARS-CoV-2 [1,2]. Most recently, the relevance of ECMO application was highlighted in the SARS-CoV-2 pandemic, representing the last therapeutic option for patients with COVID-19-related severe ARDS [2,3]. Fulminant impairment of respiratory functions in ARDS makes the use of ECMO for extracorporeal lung replacement to maintain oxygen and carbon dioxide exchange vital for the patient’s survival [4].

Despite technological progress with regard to hemocompatibility and flow optimization as well as the increasing expertise of medical staff, the rate of life-threatening complications such as infections, severe bleeding and thromboembolic events remains on a high level [5,6,7]. Thromboembolic complications in ECMO account for approx. 35% of all technical complications [5]. Considering that the recent overall mortality of ECMO-patients is approx. 46%, a mortality of approx. 23% caused by thromboembolic complications is highly alarming [8,9]. The main reason for ECMO-associated thromboembolic events and thrombosis in the membrane oxygenator (MO) is the activation of coagulation due to the contact of blood with the foreign surface area of the MO of up to 4 m^2^. Besides the risk of thromboembolic events triggered by clot migration, MO-thrombosis also reduces the effective membrane surface and restricts blood flow through the MO, resulting in insufficient gas exchange [10]. The median lifespan of an MO is only 9–12 days [11,12]. Representing a life-threatening situation, acute clot formation within the MO or pump head requires an immediate system exchange [5]. Besides specifically trained personnel needed for this emergency and high costs for system exchanges, the procedure itself always bears risks for the patient due to additional potential for thromboembolic events, bleedings, or air embolism [5]. To minimize the total number of required emergency system exchanges and subsequently turning an emergency into a better predictable situation with adequate preparation time for medical staff, early clot detection via pre-warning systems would be beneficial prior to potentially life-threatening situations for the patients.

**Figure 1:**
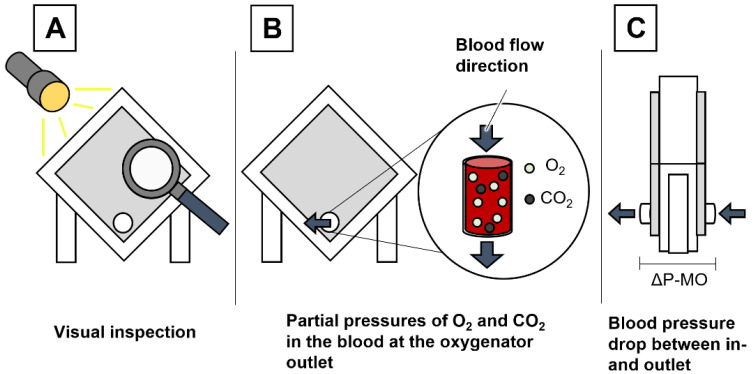
Overview of standard methods for clot detection (**A**–**C**) in a membrane oxygenator (MO) in the clinical daily routine; Visual inspection of the oxygenator (**A**), measuring the partial pressure of O_2_ and CO_2_ at the oxygenator outlet and blood pressure drop detection (**C**) as the ultimate emergency indicator for MO exchange.

Currently, various monitoring parameters for clot detection in ECMO-systems exist (Figure 1). In the clinical setting, a visual check of externally visible clot formation on the hollow fiber membrane of the MO is regularly performed (Figure 1A). Evans et al. demonstrated that an externally visible clot on the hollow fiber membrane could partially predict internal clot volume within the MO, but no significant correlation between the clot volume of external and internal clots could be shown [13]. Since fulminant clot formation on the membrane surface of the oxygenator affects gas exchange, measuring the partial pressures of oxygen and carbon dioxide in the blood at the outlet of the MO is another possible clot detection method (Figure 1B) [5]. The most common established bedside monitoring parameter in current ECMO-systems is the pressure difference across the MO (ΔP-MO), which is measured by pressure sensors upstream and downstream of the MO (Figure 1C). Clot formation within the MO obstructs blood flow, leading to an increase in ΔP-MO. According to Lubnow et al., an increase in ΔP-MO by more than 30–50% at a continuous blood flow rate requires an acute system exchange [5]. However, the value of ΔP-MO is dependent on the blood flow rate, the position of the clot inside the MO and the specific flow characteristics of the device being used [14]. Although there is a direct correlation between ΔP-MO and clot volume, the pressure values are determined via indirect measurement, which gives no direct information about the clotting status inside the hollow fiber membrane [14]. Only in already progressed clotting stages can the ΔP-MO be used to reliably detect large clot formation inside the MO [10]. Considering that the patient’s situation is already life-threatening in 1 out of 3 incidents before ΔP-MO detects complications, the lack of a reliable parameter for monitoring coagulation status and early clot detection becomes evident [5,10,15].

Bioimpedance analysis could possibly offer an additional technique to determine the coagulation status inside an MO, especially prior to fulminant clot formation using sensor-fibers integrated in the hollow fiber membrane as a prewarning system. Bioimpedance spectroscopy is a method to study the dielectric properties of biological materials to derive information on their composition. It is defined as the ability of biological tissue to resist an electric current due to the dielectric properties of the material [16,17]. The application of bioimpedance spectroscopy on blood is called dielectric blood coagulometry (DBCM). DBCM utilizes the frequency-dependent permittivity of blood, which is largely determined by the volume and concentration of red blood cells (erythrocytes). Growing blood clots incorporate a rising number of erythrocytes, increasing the volume of the clot and changing their cellular structure. In a frequency range of 100 Hz to 100 MHz, this results in an observable change in permittivity [18]. Sifuna et al. investigated not only the static but also the dynamic changes in permittivity properties in flowing blood in the frequency range around 100 KHz to 10 MHz and observed an increase in permittivity dependent on time and the coagulation progress [19]. These preceding studies suggest a correlation between coagulation and permittivity changes, thus forming the basis for the possible use of bioimpedance analysis to detect clot formation inside a MO [18,19]. Based on the results of these preceding studies that established a proof of concept for bioimpedance analysis for clot detection, an experimental dynamic test circuit was constructed integrating bioimpedance analysis and pressure difference measurement in one test circuit for the first time.

The aim of this study was to demonstrate the potential of clot detection inside a custom-made test chamber by using bioimpedance analysis and compare its predictive potential to the measured pressure difference (ΔP) across the test chamber (ΔP-TC), analogous to ΔP-MO as the gold standard for clot monitoring in clinics. As a feasibility study, this study aims to pave the way for the possible future application of bioimpedance analysis for clot detection in a MO.

## 2. Materials and Methods

### 2.1. Circuit Design

A test chamber with integrated titanium fibers for bioimpedance measurement was constructed as a proof-of-concept design, inserted into an in vitro dynamic test circuit that was roughly modeled after an extracorporeal circuit but without the gas exchange function or the enormously large foreign surface and with experimental blood volume and flow. Heparinized human blood circulated in the circuit for a maximum of 60 min or rather until fulminant clot formation was detected. For additional monitoring of coagulation, the platelet count and the β-thromboglobulin concentration (β-TG) were periodically analyzed.

**Figure 2:**
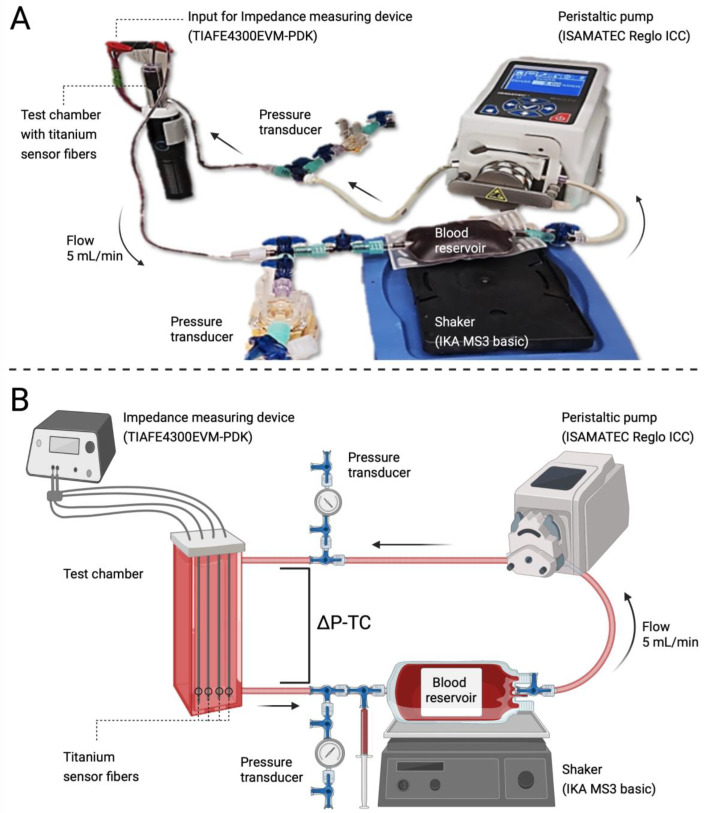
Image of the actual test circuit (**A**) and a schematic view of the test circuit (**B**). ΔP-TC: Pressure difference across the test chamber. The syringe indicates the location of blood sampling. Created with BioRender.com.

The test circuit was designed as a closed loop consisting of a blood reservoir, a test chamber and a peristaltic pump (Figure 2).

The circuit design allowed for the withdrawal of blood samples at specific time points for blood cell count and further coagulation analysis. The circuit consisted of a standard transfusion set reservoir (T3986, Fresenius Kabi AG, Bad Homburg, Germany) connected to a custom-made test chamber. Each test chamber was crafted from a semi-micro polystyrene cuvette (67.742, Sarstedt AG, Nümbrecht, Germany), which was custom modified as follows: Holes were drilled at the bottom and on the lid of the cuvette using CNC machining for precise integration and fixing of four unalloyed titanium wires (ASTM F-67 graded) for impedance measurement. Titanium as the material for the sensor fibers was chosen because of its hemocompatibility, since the material used for the sensor fibers would be in direct contact with blood, its conductivity, as this was essential for the bioimpedance measurement method used, and for its availability in fiber form, which made the integration into the test chamber and data collection possible [20,21].

The titanium wires were connected via crocodile clips to the impedance-measuring device (AFE4300, Texas Instruments Inc., Dallas, TX, USA). Polyethylene infusion lines (1.0 × 2.0 mm, Original Perfusor^®^, 8255059, B. Braun AG, Melsungen, Germany) were fixed into the test chamber’s bottom (blood inlet) and top (blood outlet).

The reservoir and test chamber were connected via multiple 3-way stopcocks (16494C, B. Braun AG, Melsungen, Germany) and PVC tubing (Tygon^®^ ST R-3607, Compagnie de Saint-Gobain S.A., Courbevoie, France) to a peristaltic pump (Ismatec 4408 Reglo ICC, Cole-Parmer GmbH, Wertheim, Germany). Pressure transducers (SP844, Memscap, Skoppum, Norway) were placed before and after the test chamber to monitor the pressure difference across the test chamber (ΔP-TC) in analogy to ΔP-MO as an indication of occlusion and thereby clot formation.

The design of the test chambers allowed us to combine impedance measurement, ΔP-TC and visualization of clot formation. For adequate visualization of clot formation after each experiment, the test chamber was installed on top of a LED flashlight (Olight M2 Warrior, Olight Technology Co., Ltd., Shenzhen, China) via a 3D-printed mount.

To reduce the risk of clot formation with the reservoir bag, the reservoir was placed on a shaker (MS3B, IKA-Werke GmbH, Staufen, Germany) set to a continuous shaking mode at 500 rpm. The entire test circuit was placed under an incubation hood (8863202, Sartorius Lab Instruments GmbH, Göttingen, Germany) to maintain a constant temperature of 37 °C. For each experiment, heparinized human whole blood was circulated while impedance and ΔP-TC changes were continuously measured inside the test chamber to detect early clot formation.

### 2.2. Blood Collection from Human Donors and Filling the Circuit

For each experiment, 50 mL of human blood was withdrawn via venipuncture into a 50 mL syringe (8728810F, B. Braun, Melsungen, Germany) primed with heparin sodium (12626723/0319, B. Braun, Melsungen, Germany). The blood was withdrawn from 20 healthy volunteers after approval from the ethical committee of the University Hospital of the RWTH Aachen University (file no EK22-355) and informed consent. According to a series of preliminary experiments, we chose 1 IU/mL blood as adequate heparinization for the control group to prevent any clot formation in the circuit, even after several hours. For the experimental group, we chose 0.5 IU/mL blood as heparinization according to the preliminary experiments, as we observed stable clot formation within 1 h of circulation. Thus, *n* = 3 control experiments were performed as a technical triplet (1 IU/mL) to receive data without any clot formation, and the remaining *n* = 17 experiments were performed with 0.5 IU/mL to enable clot formation with the circuit, depending on the individual blood constitution of the volunteers. For baseline measurements, blood was directly drawn into a 3 mL citrated sample tube (05.1165, Sarstedt AG, Nümbrecht, Germany) and a 3 mL syringe (4606027V, B. Braun, Melsungen, Germany) for subsequent cell count measurement (MEK-6500K, Nihon Kohden, Tokyo, Japan) and enzyme-linked immunosorbent assay (ELISA). The amount of heparin in the syringe and thus the grade of heparinization determined the affiliation to an experimental group. The reservoir was filled with 45 mL of the heparinized blood while air was carefully vented from the circuit. The whole test circuit was subsequently filled with blood from the reservoir by turning on the peristaltic pump. The pump flow was set to 5 mL/min for all experiments. Once the circuit was filled, avoiding entrapped air, impedance and ΔP-TC were continuously measured and recorded.

### 2.3. Pressure Measurement

The hemodynamic pressure at the inlet and the outlet of the test chamber was measured using pressure transducers (SP844, MEMSCAP S.A., Crolles, France), which were connected to the circuit via disposable domes (840-22, HJK Sensoren + Systeme GmbH & Co. KG, Merching, Germany) filled with 0.9% sodium chloride solution (3570160, B. Braun AG, Melsungen, Germany). The pressure difference across the test chamber was defined as ΔP-TC. Continuous pressure recording was performed using an amplifying bridge (ML224 Quad Bridge Amplifier, ADInstruments, Dunedin, New Zealand) and a PowerLab 16/35 data acquisition device (ADInstruments, Dundin, New Zealand). The data were monitored and captured in LabChart Pro (Version 8.1.13 2018, ADInstrments, Dunedin, New Zealand). The post-experimental data analysis was performed in Microsoft Excel (Microsoft 365 MSO, Version 2202 Build 16.0.14931.20118 32 Bit, Microsoft Corp., Redmond, WA, USA).

### 2.4. Fundamentals of Bioimpedance Measurement

Biological tissues are in most cases conductive dielectrics, which is also true for blood. The charge carriers that conduct the electric current through the material are mainly ions, but their mobility is limited [16].

**Figure 3:**
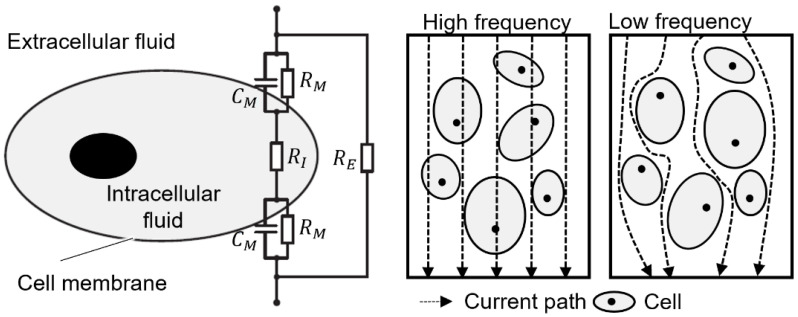
Electrical equivalent circuit of a cell (**left**) and current paths through cell suspensions (**right**).

In low frequency ranges, the cell membrane delays the conduction of the electric current by briefly storing charges as in a capacitor. This stored charge counteracts the external current flow when the direction changes. At low frequencies, this prevents the current from flowing through the cell. The intracellular fluid is not passed through, or only partially, as shown in Figure 3 on the right for low frequencies [22,23].

For biological environments, three ranges can be distinguished based on the polarization mechanisms and current paths present. The effects dominate in narrow, specific frequency ranges called α-, β- and γ-dispersion. The frequency at which the change occurs and the extent of the dispersion allow conclusions to be drawn about the material under investigation via the impedance or the electrical properties it contains. The changes are particularly evident in the measured values of permittivity ε and specific conductivity σ. These two quantities are material-specific characteristic values when an electric field is applied [24,25,26].

### 2.5. Bioimpedance Measurement

The impedance measurements were performed in the single-frequency mode of the measuring device. The AFE4300EVM-PDK (Texas Instruments Inc., Dallas, TX, USA) was used as an analog front-end capable of bioimpedance measurement. The constant measuring frequency was 50 kHz, and 128 values per second were recorded by the internal circuitry. The values were recorded in codes, the unit specific to the measuring device, which had to be converted into an impedance for the analysis.

**Figure 4:**
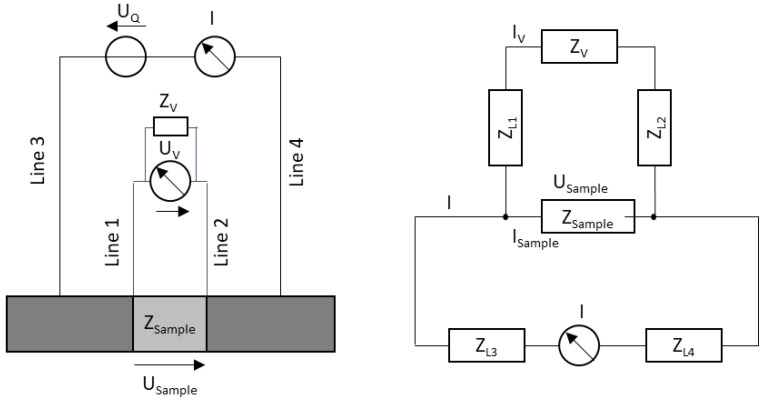
Structure of the four-wire measurement method (**left**) and equivalent circuit (**right**).

Four unalloyed medical grade 1 titanium wires (Ø 0.20 mm, ASTM-F67-certified) were integrated into the test chamber that was used in this study. The measurement of an impedance using four electrodes is called four-terminal sensing or the Kelvin method. The electrodes were used in pairs, as shown in Figure 4.

An alternating current I with constant amplitude was impressed on the outer of the two electrode pairs (line 3 and 4) to generate an electric field. The inner pair of electrodes (line 1 and 2) measured the resulting voltage drop USample between the voltage contacts due to the applied field via a voltmeter. From excitation current I, electrode spacing and sample cross-section, the impedance of the sample between the inner electrodes could be calculated via Ohm’s law.

In accordance with the device’s user guide, the device was calibrated using two reference resistors measured as 10.2 Ohm [Ω] and 218.5 Ohm [Ω]. Since the voltage measured by the device was linear to the magnitude of impedance, the measured voltages of the two reference resistors were used to calculate a 2-point linear equation, allowing us to accurately convert registered voltage into bioimpedance.

The data were monitored and obtained using the software provided by the manufacturer (AFE4300 Device GUI, Version 1.16, Texas Instruments Inc., Dallas, TX, USA). A post-experiment workup for analysis was conducted in Excel (Microsoft 365 MSO, Version 2202 Build 16.0.14931.20118 32 Bit, Microsoft Corp., Redmond, WA, USA).

### 2.6. Experimental Groups

Two experimental groups were defined by the initial grade of heparinization. Preliminary experiments with various grades of heparinization were conducted to establish a setup that would provide sufficient anticoagulation to prevent instant clotting but still allow clotting within the experiment’s timespan of 60 min. A heparinization of 0.5 IU/mL provided reliable clot formation within the experiment’s time span and was therefore defined as the “Clotting group”. The control group was heparinized with 1 IU/mL to ensure experiments without clot formation.

### 2.7. Experiment Duration and Criteria for Experiment Termination

A total duration of 60 min for each experiment was predefined as the time window for clotting, according to preliminary experiments. However, as soon as a persistent relative spike of ΔP-TC > 50% as an indicator for clot formation inside the test chamber was observed, the experiment was terminated and the timepoint was recorded as the end point. This cutoff was chosen in accordance with the criteria for an acute MO change in a clinical setting regarding the increase in ΔP-MO [6]. This end point was defined via a significant ΔP-TC increase that indicates a relevant occlusion, with the subsequent compromising hemodynamics caused by clot formation. As soon as these conditions occurred, one final blood sample was drawn from the circuit and the experiment was terminated. Immediately after termination of the experiment, the test chamber was flushed using 0.9% sodium chloride solution and subsequently transilluminated from the bottom to the top using a flashlight for clot visualization.

### 2.8. Blood Sampling and Analysis

Every 10 min during the experiments, blood samples were drawn into a 1.1 mL serum tube (41.15000.05, Sarstedt, Nümbrecht, Germany) and a 1 mL citrated tube (41.1506, Sarstedt, Nümbrecht, Germany). The immediate analysis comprised a hemogram using an automated cell counter (MEK-6500K, Nihon Kohden, Tokyo, Japan).

The blood samples were centrifuged at 2000 g for 10 min at room temperature (21 °C) and aliquoted to obtain blood plasma and serum samples, which were then stored at −80 °C. The blood serum samples were used to conduct an enzyme-linked immunoassay (SEA370Hu 96 Kit for Beta-Thromboglobulin, Cloud-Clone Corp., Katy, TX, USA) for in vitro quantitative measurement of β-TG for additional monitoring of the coagulation status.

### 2.9. Statistical Analysis

For each experiment, the 20 min timeframe prior to the significant ΔP-TC increase was analyzed. Impedance and pressure data were tested for normal distribution using the Anderson–Darling test. Since the pressure data were normally distributed, a one-way-ANOVA with repeated measures was performed. Due to lognormal distribution of the bioimpedance data, a nonparametric Friedman test was performed. For both pressure and impedance data in the clotting group, the mean values of a 30 s interval within the 20 min timeframe prior to the significant ΔP-TC increase were compared to the baseline timepoint. The differences were considered as significant if the calculated *p*-value was below 0.05.

In the control group, the baseline values were compared to every following one-minute value for 60 min. The same statistical tests for pressure and impedance as in the clotting group were used. A *p*-value below 0.05 was considered significant.

The ELISA-data were statistically analyzed using a Two-way-ANOVA (Šídák’s multiple comparisons test) comparing the concentration of β-TG every 10 min in the clotting group with the control group. The same statistical test was used for the platelet count data. A *p*-value below 0.05 was considered significant.

GraphPad Prism software (Version 9.3.1, GraphPad Software, San Diego, CA, USA) was used for statistical analysis and graph design.

## 3. Results

### 3.1. Blood Clot Formation

**Figure 5:**
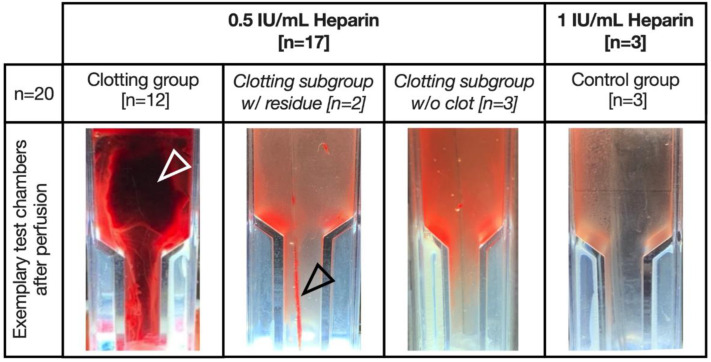
Exemplary images of test chambers after flushing with sodium chloride solution. White arrow: fulminant clot; black arrow: macroscopic residue on the sensor fibers.

Variations in blood clot formation in the distinct experimental groups and exemplary test chambers after perfusion are shown in Figure 5.

Occurrence of clot formation in the clotting group (0.5 IU/mL heparin) was not homogenous. Fulminant clot formation occurred in 12 out of 17 experiments (Figure 5, white arrow).

However, in five experiments, no fulminant clot formation was observed, though two of these experiments exhibited subtle residue on the sensor fibers (Figure 5, black arrow, clotting subgroup w/residue). Three of these five experiments did not show clot formation at all (clotting subgroup w/o clot).

In comparison, in the control group (1 IU/mL heparin), no clot formation in the test chamber occurred after 60 min.

### 3.2. Pressure and Impedance Measurement

**Figure 6:**
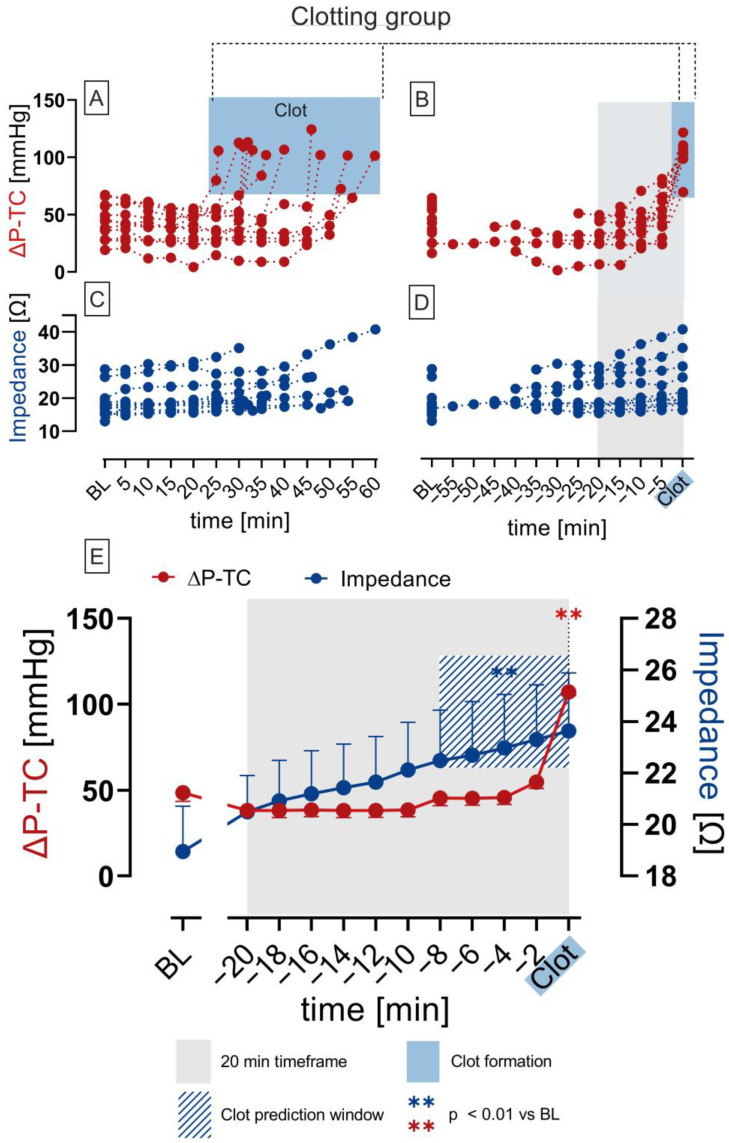
Impedance and pressure difference measurements (ΔP-TC) over time of clotting group (0.5 IU/mL heparin) with fulminant clot formation (*n* = 12). Overview of ΔP-TC (**A**) and impedance (**C**) of each experiment starting from baseline to termination. ΔP-TC (**B**) and impedance (**D**) arranged and regarded 20 min retrospectively (grey box) from the endpoint of the experiments with fulminant clot formation (blue box). Mean ± SEM of ΔP-TC and impedance in the 20 min timeframe prior to fulminant clot formation. Significant increase in impedance 8 min before significant increase in ΔP-TC (blue stripes window; (**E**)); ANOVA; ** *p* < 0.01 vs. baseline (BL).

Since time points of fulminant clot formation varied in each experiment (Figure 6A,C), the timeframe 20 min before fulminant clot formation was investigated and compared (Figure 6B,D). In the clotting group with fulminant clot formation, impedance measurement significantly increased 8 min prior to significant ΔP-TC increase and indicated clot formation, entailing the termination of the experiments (Figure 6E) (** *p* < 0.01).

**Figure 7:**
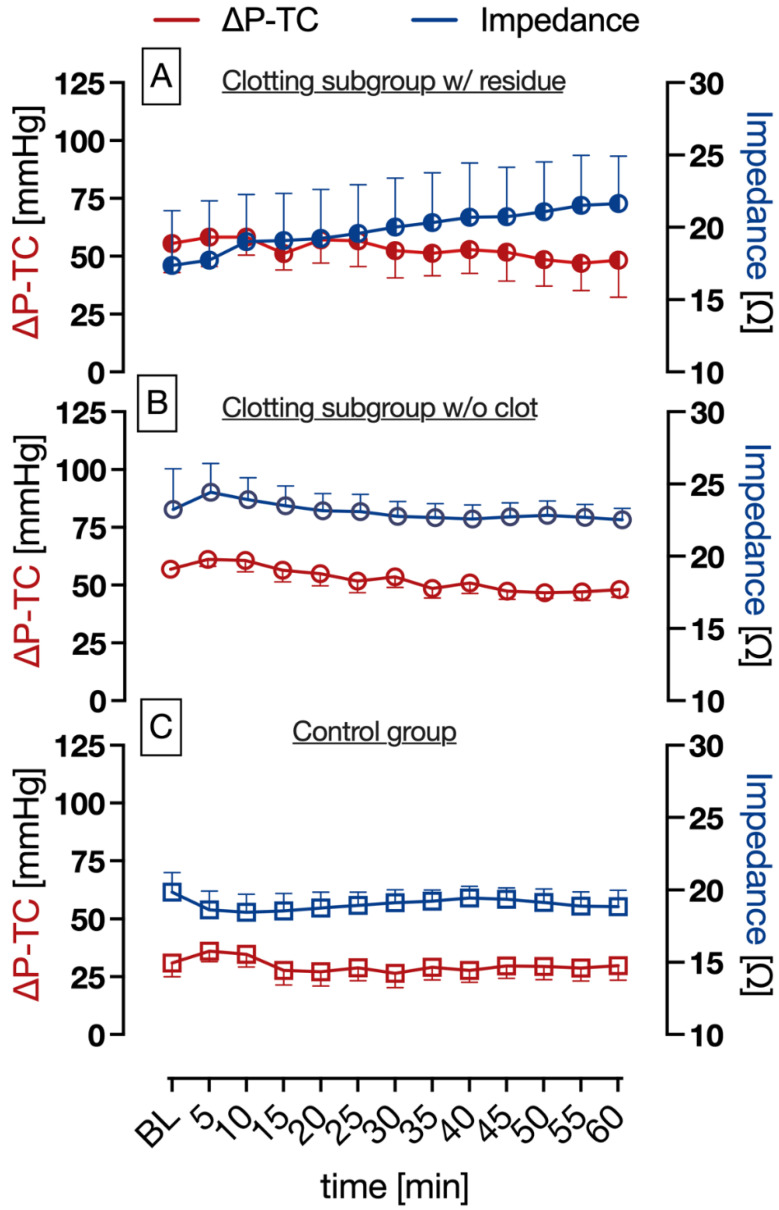
Impedance and pressure difference measurements (ΔP-TC) over time. (**A**): clotting subgroup w/residues on impedance-measuring fibers (*n* = 2), subtle, non-significant increase in impedance and no significant increase in ΔP-TC. (**B**): clotting subgroup w/o clot formation (*n* = 3), no significant increase in impedance and ΔP-TC. (**C**): control group (*n* = 3), no significant increase in impedance and ΔP-TC; ANOVA; mean ± SEM.

In the clotting subgroup w/residue on the sensor fibers, no significant changes in ΔP-TC and impedance were measured (Figure 7A, *p* > 0.05). Nevertheless, the increase in impedance in this subgroup was not statistically significant, yet a subtle increase in impedance was still observed (Figure 7A). In the clotting subgroup w/o clot formation, no significant changes in impedance and ΔP-TC were observed (Figure 7B *p* > 0.05).

In the control group (1 IU/mL heparin), no statistically significant changes in ΔP-TC and impedance measurements were observed over a runtime of 60 min (Figure 7C; *p* > 0.05).

### 3.3. Platelet Count and β-Thromboglobulin

**Figure 8:**
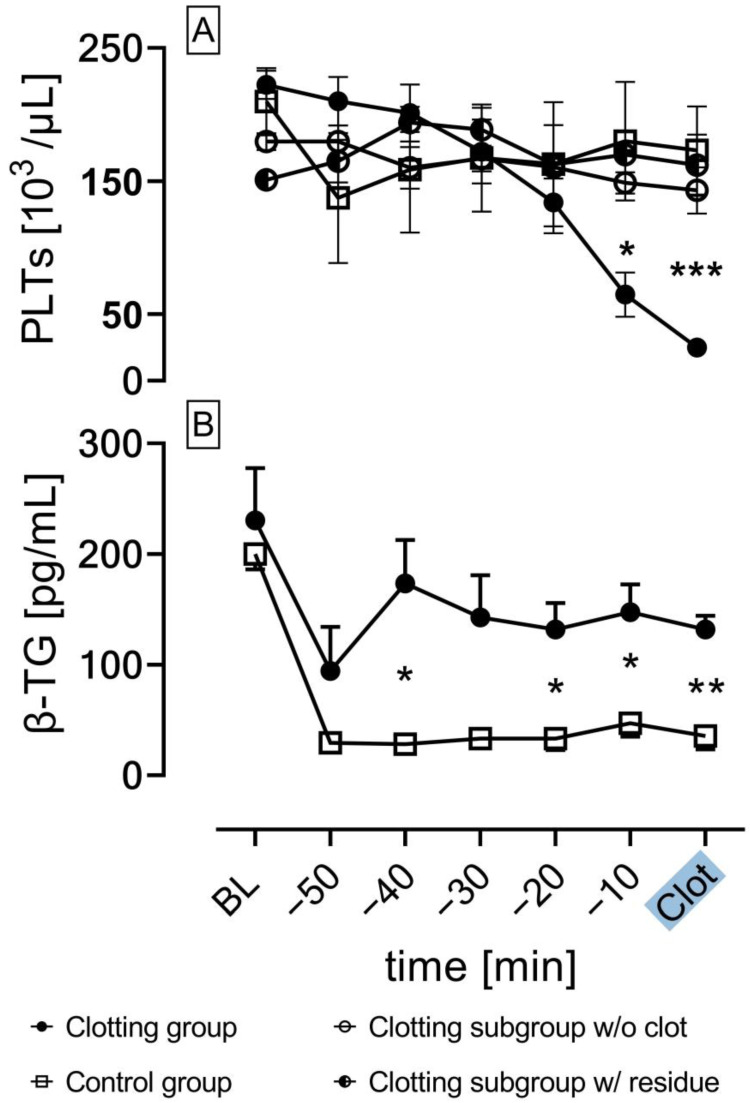
(**A**): Platelet count (PLTs) over time in the clotting group (0.5 IU/mL; *n* = 12), control group (1 IU/mL; *n* = 3), clotting subgroup w/o clot formation (0.5 IU/mL; *n* = 13) and the clotting subgroup w/residues (0.5 IU/mL; *n* = 2); significant decrease in the number of platelets in the clotting group with fulminant clot formation; (**B**): β-thromboglobulin concentration (β-TG) over time in the clotting group with fulminant clot formation (*n* = 12) and control group (*n* = 3); ANOVA; mean ± SEM; * *p* < 0.05; ** *p* < 0.01; *** *p* < 0.001.

The platelet count of the clotting group experiments with fulminant clot formation (0.5 IU/mL; *n* = 12) was compared to that of the control group (1 IU/mL; *n* = 3) and the clotting subgroups w/o clot formation (0.5 IU/mL; *n* = 3) and w/residue (0.5 IU/mL; *n* = 2) (Figure 8A). The platelet count in the clotting group significantly decreased 10 min (t = −10 min; * *p* < 0.05 vs. Control group) before the experiment was terminated (t = Clot; * *p* < 0.05 vs. clotting subgroup w/o clot formation, subgroup w/residue; *** *p* < 0.001 vs. Control group). No significant decrease in the platelet count was observed in experiments without fulminant clot formation.

The concentration of β-TG as a marker for platelet activation was measured in the extracted serum blood samples using ELISA. Figure 8B compares the β-TG concentration of the clotting group experiments with fulminant clot formation (*n* = 12) with the control group (*n* = 3). In the clotting group, a significant increase in β-TG concentration was measured after 20 min as opposed to the control group (* *p* < 0.05) and persisted until fulminant clot formation terminated the experiments. No significant increase in β-TG concentration was observed in the control group (*p* > 0.05).

## 4. Discussion

The aim of this study was to investigate the capability of bioimpedance analysis regarding blood clot detection inside a test chamber connected to an in vitro test circuit perfused with human blood for a maximum duration of 60 min. The coagulation status inside the test chamber was monitored via bioimpedance measurements and compared to the measurement of the pressure difference across the test chamber (ΔP-TC), in analogy to the pressure difference across the MO (ΔP-MO) that represents the current clinical gold standard for blood clot detection. This experimental test setup of a biosensor consisting of a test-chamber and circuit allowed a direct and continuous comparison between the clot detection capabilities of bioimpedance analysis and pressure difference measurement.

### 4.1. Bioimpedance Analysis and Pressure Difference Measurement (ΔP-TC)

This study demonstrates that bioimpedance analysis can reliably indicate clot formation inside a blood-perfused test chamber: When bioimpedance significantly increased, a blood clot could be detected. When there were no changes in bioimpedance, no clot formation was present. Additionally, the bioimpedance signal increased significantly earlier (8 min) in comparison to the significant increase in ΔP-TC. This means that bioimpedance analysis is not only capable as a clot detector, but also as a predictor, in contrast to ΔP-TC, which only acts as a detector if extensive clot formation occurs and the flow path is constricted (Figure 6E). These in vitro findings indicate that bioimpedance analysis could be more sensitive in detecting blood clots from the very beginning of their formation on foreign surfaces as present in our test chamber, whereas ΔP-TC, analogous to ΔP-MO, the gold standard used in clinics to detect a clot within a MO, detects a clot at a later state, when the flow path is already occluded by a fulminant clot. Even marginal residues of blood cells on the biosensor resulted in a change in the bioimpedance signal.

These findings are supported by the experimental group without fulminant clot formation but still having slight residue on the sensor fibers (clotting subgroup w/residue), which could be responsible for the subtle but not significant increase in bioimpedance measurement (Figure 7A).

Furthermore, the results of the control group (Figure 7C) and the experiments in the clotting group without clot formation (Figure 7B) show that neither an increase in bioimpedance or ΔP-TC was observed when clot formation was absent. Thus, we could clearly evidence that significant increases in bioimpedance and ΔP-TC were associated with clot formation, but bioimpedance had an additional predictive effect of 8 min.

The utilization of dielectric blood coagulometry (DBCM) to analyze the coagulation status of blood has been previously investigated by various groups. Hayashi et al. investigated the dielectric response of static blood coagulation with human blood and established a proof of concept that DBCM can sensitively measure blood coagulation by detecting an increase in permittivity caused by red blood cell aggregation [18]. Sifuna et al. investigated the utilization of DBCM for permittivity-based clot monitoring by integrating a connector sensor into a dynamic circuit in which swine blood circulated and compared its clot detecting capabilities with optical coherence tomography imaging [19]. The main difference to our study regarding the experimental setup was that we directly compared impedance analysis with pressure difference measurement for the first time. Additionally, our setting was not established for static measurement, but under flow conditions, even though the conditions were not clinically relevant. Though modern ECMO-systems are capable of continuous ΔP-MO acquisition as the standard parameter for blood clot monitoring, a more sensitive and continuous analysis method also working under flow conditions might add value to the clinical standard. Additionally, bioimpedance measurement and pressure difference are convenient and inexpensive parameters compared to, for instance, optical coherence tomography [27].

This feasibility study shows that bioimpedance analysis is not only capable of reliably detecting clot formation in an in vitro test circuit and test chamber, but that it outperforms pressure difference measurement as well, thus paving the way for future studies that aim to investigate the integration of bioimpedance analysis into an ECMO setting. The integration of electrodes for impedance measurement into hollow fiber membranes of MOs is a challenge to strive for that would enable the establishment of a new monitoring parameter that could allow not only the early detection of critical clot formation but also a more precise localization of clots by integrating multiple electrodes across the oxygenator membrane. Since clot formation in the MO is considered more or less critical depending on localization, precise localization of clots could allow a risk-based assessment of when to perform an acute system exchange that would potentially spare unnecessary system exchanges with all the associated costs and resources [10].

### 4.2. Coagulation Parameters

The experimental setup in combination with the coagulation protocol ensured spontaneous clot formation in the test chamber, induced by continuous exposure of blood to the foreign surface of the test circuit [28]. The circulating blood in an extracorporeal circuit is constantly in contact with foreign materials that lead to immediate protein adsorption on the artificial surface, especially albumin and fibrinogen. Furthermore, turbulences and shear stress generated by the circuit pump damage cells, which release von Willebrand factor, resulting in platelet activation and adhesion to the artificial surface. Subsequently, this leads to platelet aggregation via expressed GPIIb/IIIa receptors on the platelet surface that bind to adsorbed proteins such as fibrinogen. The increasing platelet consumption caused by the progressing coagulation results in a significant platelet drop, which highlights its importance as a clot monitoring parameter in ECMO-systems [29,30]. Therefore, the significant decrease in platelets (Figure 8A) in the clotting group with fulminant clot formation is caused by platelet aggregation in the growing clot, since aggregated platelets are not measured in the platelet count. The drop in platelets was significant approximately 10 min before the experiments reached their endpoint. In contrast, the control group and the subgroups of the clotting groups with no clot formation demonstrated no significant platelet drop.

β-TG is a protein that is released from alpha granules of activated platelets and can be used as a reliable marker for platelet activity [31]. Figure 8B shows a significant increase in β-TG in the clotting group with fulminant clot formation compared to the control group without clot formation, thus indicating platelet activation. The higher heparin concentration in the control group affects platelet function via thrombin inhibition, as thrombin is a potent platelet activator [32]. Moreover, heparin positively modulates the interactions of von Willebrand factor with the platelet GPIb/V/IX complex, also leading to the inhibition of platelet aggregation [33]. In previous in vitro studies, β-TG was a reliable marker for platelet activation and subsequent clot formation, matching the findings of this study [34]. The initial higher values of β-TG in both the clotting group and the control group could be explained by the fact that the baseline values were measured from the samples directly drawn from the volunteers, thus representing the in vivo concentration. Interestingly, 3 out of 17 experiments in the clotting group were categorized to the subgroup w/o clot formation marked by no clot formation, no changes in impedance and no changes in ΔP-TC. A plausible explanation for the incidence of this subgroup could be the interindividual differences in the coagulation profile of probands and the individual effect of anticoagulant drugs such as heparin [35].

### 4.3. Limitations

One limitation of our study is the size and setup of our test circuit, since it is a highly simplified model of an extracorporeal circuit. Our test circuit lacks the properties of an ECMO device with its gas exchange membrane, its priming volume, and its usual much higher blood flow rate. The larger inlet and outlet of an ECMO occlude by clot formation at a much lower rate than in our miniaturized test circuit. In our test circuit, the test chamber was more susceptible to occlusion by blood clots compared to MO. Furthermore, studies have shown that hematocrit also affects bioimpedance analysis [18,19]. Another limitation of this study is that the test chambers containing the titanium fibers for impedance measurement were all made by hand; therefore, the effects of contamination and residues on the fibers cannot be ruled out entirely.

## 5. Conclusions

In conclusion, this study shows that clot detection via bioimpedance analysis under flow conditions in a blood-perfused test chamber is feasible. Additionally, bioimpedance analysis, in comparison to pressure difference measurement, the gold standard for clot monitoring in intensive care unit daily routine, demonstrated a significantly earlier clot detection and therefore a potential to act as a clot predictor, not only a detector.

However, due to the highly simplified experimental setup and the lack of ECMO properties in terms of a gas exchange membrane, blood volume and flow, the transferability of results to actual MO is restricted. Therefore, further investigation evaluating bioimpedance analysis in miniaturized MO with integrated hollow fiber membranes is essential.

## Data Availability

The datasets generated and/or analyzed during the current study are available from the corresponding author upon reasonable request.
